# NK cells and multiple myeloma-associated endothelial cells: molecular interactions and influence of IL-27

**DOI:** 10.18632/oncotarget.17070

**Published:** 2017-04-12

**Authors:** Alessandra Dondero, Beatrice Casu, Francesca Bellora, Angelo Vacca, Annunziata De Luisi, Maria Antonia Frassanito, Claudia Cantoni, Silvia Gaggero, Daniel Olive, Alessandro Moretta, Cristina Bottino, Roberta Castriconi

**Affiliations:** ^1^ Department of Experimental Medicine (DIMES), University of Genova, 16132 Genova, Italy; ^2^ Department of Biomedical Sciences and Human Oncology, University of Bari, 70124 Bari, Italy; ^3^ Istituto Giannina Gaslini, 16147 Genova, Italy; ^4^ Center of Excellence for Biomedical Research (CEBR), University of Genova, 16132 Genova, Italy; ^5^ U1068, CRCM, Immunity and Cancer, INSERM, 13009 Marseille, France

**Keywords:** multiple myeloma, endothelial cells, NK cells, IL-27, PD-Ls

## Abstract

Angiogenesis represents a hallmark of tumor progression in Multiple Myeloma (MM), a still incurable malignancy. Here we analyzed the activity of cytokine-stimulated NK cells against tumor-associated endothelial cells isolated from bone marrow aspirates of MM patients with active disease (MMECs). We show that NK cells activated with optimal doses of IL-15 killed MMECs thanks to the concerted action of multiple activating receptors. In particular, according to the high expression of PVR and Nectin-2 on MMECs, DNAM-1 actively participated in target recognition. Interestingly, in MMECs the surface density of PVR was significantly higher than that detected in endothelium from patients with MM in complete remission or with monoclonal gammopathy of undetermined significance (MGUS). Importantly, IL-27, which unlike IL-15 does not display pro-angiogenic properties, maintained or increased the NK cell functions induced by suboptimal concentrations of IL-15. NK cell properties included killing of MMECs, IFN-γ production as well as a peculiar increase of NKp46 expression on NK cell surface. Finally, IL-27 showed a striking capability of up-regulating the expression of PD-L2 and HLA-I on tumor endothelium, whereas it did not modify that of PD-L1 and HLA-II.

Our results suggest that cytokine-activated endogenous or adoptively transferred NK cells might support conventional therapies improving the outcome of MM patients.

## INTRODUCTION

In the last decades important advancements in the treatment of different malignances have led to significant improvement in patients’ survival. A variety of therapies are currently available including those targeting specific pathways involved in the growth and survival of cancer cells or tumor-associated stromal cells such as endothelial cells. A pro-tumoral crosstalk between cancer and endothelial cells occurs and is essential for tumor growth. In multiple myeloma (MM), an incurable malignancy of monoclonal plasma cells [1], angiogenesis represents a hallmark of tumor progression and anti-VEGF drugs, alone or in combination with other agents, are currently used. In some patients however, available therapeutic approaches resulted in limited benefits and in a short-lasting tumor regression. Thus, many efforts have been made to evaluate additional therapeutic protocols aimed at obtaining a more durable tumor control in different malignances including MM. New strategies consist of immunotherapeutic approaches including the strengthening of the function of endogenous Natural Killer (NK) cells or the adoptive transfer of “armed” or activated NK cells [2–5]. These cells are cytotoxic members of the Innate Lymphoid Cell (ILCs) family [6, 7] and have been shown to play a crucial role in tumor surveillance also due to their capability of killing tumor cells including those with stem cell like properties [8–11]. The mechanisms allowing the NK-mediated recognition of tumor cells have been largely clarified and consist of the cooperation of different triggering receptors that are engaged by specific ligands upregulated or de novo expressed on transformed cells [3, 11, 12]. Activating NK receptors are represented by NKp46 (CD335), NKp30 (CD337) and NKp44 (CD336) (collectively termed natural cytotoxicity receptors, NCR), DNAM-1 (CD226) and NKG2D (CD314) [3, 13]. Although viral glycoproteins have been identified as ligands for NCR, their cellular ligands are not fully defined [14]. Two molecules, the mixed-lineage leukemia (MLL5) [15] and B7-H6 [16] have been found to be expressed on a wide panel of tumors [17] and represent cellular surface ligands of NKp44 and NKp30, respectively. It is of note however, that different evidences suggest that NKp30 would be able to recognize additional, still undefined, tumor-associated ligand(s). NKG2D recognizes MICA/B and ULBPs [12] stress inducible molecules de-novo expressed after tumor transformation and virus infection. DNAM-1 binds PVR (CD155) and Nectin-2 [18], two members of the Nectin family that are also recognized by Tactile [19] and TIGIT [20]. PVR and Nectin-2 are over-expressed in tumors of different histotype and their interaction with DNAM-1 is non-redundant and crucial to obtain an efficient tumor cell killing [21–24]. Accordingly, different studies highlighted the importance of DNAM-1/ligands interactions in the establishment of the activating immunological synapse that allows tumor recognition by both NK and T cells [25–28].

The possible effect of NK-cell based therapeutic approaches in killing of tumor-associated endothelial cells with the consequent reduction of the vascular network remains to be determined.

In the present study we analyzed the NK cell activity against tumor-associated endothelial cells (EC) isolated from bone marrow aspirates of MM patients in active phase (MMECs). We evaluated the susceptibility of MMECs to killing mediated by IL-15-stimulated NK cells and dissected the molecular interactions occurring between effector and target cells. Moreover, we investigated the immunostimulatory effects of IL-27 [29] that, unlike IL-15, does not display pro-angiogenic properties [30, 31]. Finally, considering that NK cell activation might result *in vivo* in a cytokine storm responsible for the activation of immune checkpoints, [32, 33] we analyzed in MMECs the constitutive and cytokine-induced surface expression of Programmed Death Ligands (PD-Ls) and HLA class I and II [34–36].

## RESULTS

### DNAM-1 actively participates to the killing of MMECs mediated by rIL-15-activated NK cells

**Figure 1:**
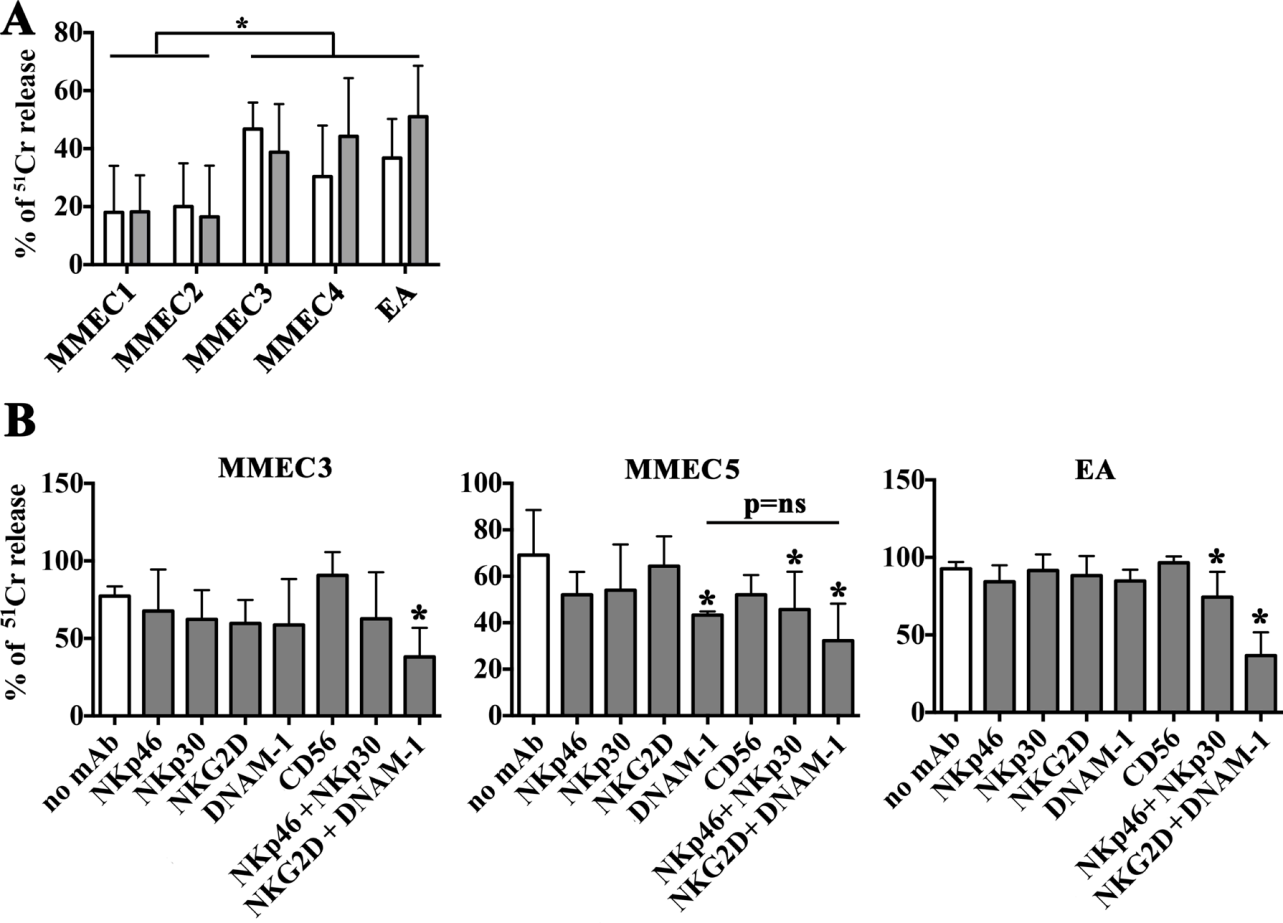
Susceptibility of MMECs to NK-mediated killing and activating receptors involved (**A**) PBMCs from 3 healthy donors were stimulated with rIL-15 (20 ng/ml) and analyzed for their cytolytic activity (^51^Cr release assay) against MMECs derived from 4 different patients (MMEC1-4), (E:T ratio 80:1) in the absence (white bars) or in the presence of anti-HLA-I mAbs (gray bars). Mean (3 healthy donors in duplicate), 95% confidence intervals and significance are indicated. *p < 0.05. (**B**) IL-15 activated NK cell populations were analyzed for their cytolytic activity (^51^Cr release assay) against MMECs and EA cell line (E:T ratio 20:1) in the absence (white bars) or in the presence of mAbs (10 μg/ml) specific for the indicated activating NK receptors used alone or in combination. Mean (3 healthy donors in duplicate), 95% confidence intervals and significance are indicated. *p < 0.05.

Tumor-associated endothelial cells were isolated from bone marrow (BM) aspirates of nine Multiple Myeloma Patients in active phase (Table 1) [37]. MMECs were analyzed for the susceptibility to lysis mediated by peripheral blood mononuclear cells (PBMCs) of healthy donors activated with optimal doses of rIL-15 (20 ng/ml) (Figure 1A). Overall, activated PBMCs killed the MMECs analyzed and HLA class I molecules had a poor protective role as demonstrated by the lack of significant differences observed in the presence of the anti-HLA-I mAb (Figure 1A). It is of note however that a certain degree of heterogeneity in the susceptibility of MMECs to activated PMBCs could be appreciated. Indeed, MMEC3 and MMEC4 showed a susceptibility to lysis comparable to that of EA, a prototypic tumor endothelial cell line used as control, whereas MMEC1 and MMEC2 were more resistant (Figure 1A).

**Table 1:** Endothelial cells analyzed in the study

Endothelial cells	Disease	Phase	Age	Gender
MMEC1	MM	Active	55	Male
MMEC2	MM	Active	63	Female
MMEC3	MM	Active	70	Male
MMEC4	MM	Active	67	Female
MMEC5	MM	Active	73	Female
MMEC6	MM	Active	55	Male
MMEC7	MM	Active	65	Male
MMEC8	MM	Active	60	Female
MMEC9	MM	Active	63	Female
cr-MMEC	MM	Complete remission	71	Male
MGEC1	MGUS		47	Male
MGEC2	MGUS		59	Male
MGEC3	MGUS		61	Female
MGEC4	MGUS		75	Female
MGEC5	MGUS		53	Male
MGEC6	MGUS		75	Male
MGEC7	MGUS		64	Male
IDAEC	IDA		56	Female

The endothelial cells analyzed in this study were derived from bone marrow aspirates of patients with Multiple Myeloma in active phase (MMEC1-9) or complete remission (cr-MMEC), patients with monoclonal gammopathy of undetermined significance (MGEC1-7) and a patient with anemia due to iron deficiency (IDAEC).

Assuming a predominant role of NK lymphocytes in the killing of MMECs by rIL-15 activated PBMCs, we analyzed the susceptibility of MMECs to lysis mediated by highly purified activated NK cells (Figure 1B). Moreover, in order to analyze the possible contribution of the different activating NK receptors in the recognition of MMECs, cytolytic assays were performed in the presence of mAbs able to specifically disrupt the interactions between the receptors (on NK cells) and their ligands (on target cells). Similar to EA, MMECs were highly susceptible to killing mediated by rIL-15 activated NK cells, a process that depended on the cooperation of various activating receptors (Figure 1B). In particular, NKG2D and DNAM-1 contributed to the killing of MMEC3 and a significant inhibition of lysis was observed only after the combined mAb-mediated masking of both molecules. NKG2D was not involved in MMEC5 recognition, whereas DNAM-1 played a major role in the NK-mediated cytotoxicity, as its mAb-mediated masking resulted in a significant reduction of lysis. Moreover, mAb-mediated masking of NKp30 and NKp46 significantly reduced the lysis demonstrating the involvement of these receptors in killing of MMEC5 (Figure 1B). The NK-mediated recognition of EA cells involved the four different activating receptors thus recapitulating what observed in endothelial cells derived from MM patients. A similar scenario was observed using endothelial cells obtained from patients with monoclonal gammopathy of undetermined significance (MGECs). In these experiments we used the CD107a assay that was more suitable to preserve the viability of target cells. As shown in Supplementary Figure 1, rIL-15 stimulated NK cells degranulated in the presence of MGECs (and in the presence of EA, used as control) and DNAM-1, NKG2D, NKp30 and NKp46 receptors clearly cooperated in the process.

### MMECs and EA cell line express the ligands of DNAM-1 activating receptor

MMECs were analyzed for the surface expression of the ligands of activating receptors known to regulate NK cell functions including cytolytic activity. The gating strategy is shown in Supplementary Figure 2. For comparison, the analysis was performed on endothelial cells derived from BM of patients with MM in complete remission (cr-MMEC), monoclonal gammopathy of undetermined significance (MGEC 1-5) or anemia due to iron deficiency (IDAEC).

**Figure 2:**
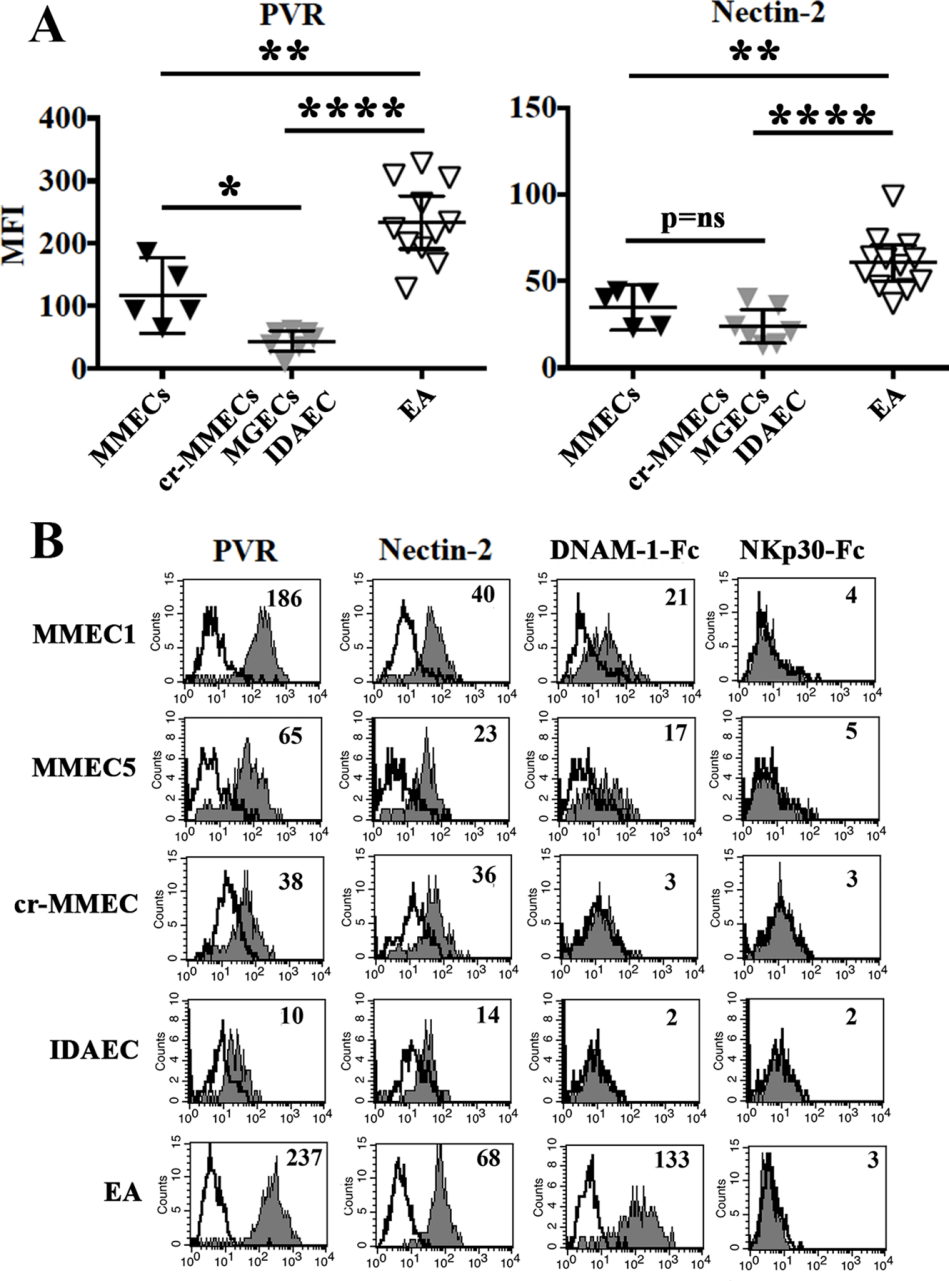
PVR and nectin-2 expression in MMECs and normal endothelial cells (**A**) MMECs (black triangles), cr-MMEC, MGECs, IDAEC (gray triangles) and EA (white triangles) were analyzed by flow cytometry for the expression of PVR and Nectin-2. Mean and 95% confidence intervals are indicated. *p < 0.05, **p < 0.0001, ****p < 0.001, *p* = ns means not significant. (**B**) Representative cytofluorimetric analysis of PVR, Nectin-2, DNAM-1-Fc and NKp30-Fc staining in MMECs, cr-MMEC, IDAEC and EA. White profiles refer to cells incubated with isotype-matched mAbs. Value inside each histogram indicates the Median Fluorescence Intensity (MFI).

In all cells analyzed NKG2D-ligands were either undetectable or expressed at very low levels (Table 2). In particular, according to the involvement of NKG2D in NK-mediated lysis (see Figure 1B) MMEC3 expressed MICA, ULBP-2 and ULBP-3. The latter two ligands were also detected in the EA cell line whereas MMEC5, which was killed in an NKG2D-independent manner, did not express any of the NKG2D ligands analyzed. All endothelial cells analyzed expressed good levels of PVR and Nectin-2 (Table 2), ligands of DNAM-1 receptor involved in the killing of MMECs and EA (see Figure 1B). Interestingly, in MMECs and EA the surface density of PVR was significantly higher than in cr-MMEC, MGECs and IDAEC (Figure 2A). Accordingly, MMECs and EA but not the other endothelial cells analyzed were stained by the DNAM-1-Fc soluble receptor (Figure 2B).

**Table 2:** NKG2D and DNAM-1 ligands expression in endothelial cells

	ULBP-1	ULBP-2	ULBP-3	ULBP-4	MICA	PVR	Nectin-2
**MMEC1**	**(−)**	10	11	**(−)**	**(−)**	186	40
**MMEC2**	**(−)**	ND	ND	ND	ND	147	43
**MMEC3**	**(−)**	7	13	**(−)**	12	93	44
**MMEC4**	**(−)**	**(−)**	10	**(−)**	ND	93	24
**MMEC5**	**(−)**	**(−)**	(−)	**(−)**	**(−)**	65	23
**cr-MMEC**	**(−)**	**(−)**	(−)	**(−)**	**(−)**	38	36
**MGEC1**	**(−)**	12	(−)	**(−)**	**(−)**	57	24
**MGEC2**	ND	**(−)**	9	ND	ND	35	21
**MGEC3**	**(−)**	10	10	13	**(−)**	49	19
**MGEC4**	**(−)**	**(−)**	**(−)**	6	**(−)**	61	40
**MGEC5**	**(−)**	6	**(−)**	ND	ND	57	13
**IDAEC**	ND	(−)	**(−)**	ND	ND	10	14
**EA**	**(−)**	12	24	**(−)**	**(−)**	257	57

Endothelial cells used in this study were analyzed by flow cytometry for the expression of the indicated ligands. Values indicate the MFI. Undetectable = (–), ND = not determined. For EA cell line values represent the mean of 10 different experiments.

In spite of the clear involvement of NKp30 in the NK-mediated lysis of some MMECs and EA cells, none of the endothelial cells analyzed was stained by the NKp30-Fc soluble receptor (Figure 2B), which efficiently bound the B7-H6 specific ligand on B7-H6+ cell transfectants and K562 cell line (Supplementary Figure 3A). EA cell line was characterized by a very low level of B7-H6 transcript, comparable to that detected in immature Dendritic cells (iDC), whose interaction with NK cells is mostly NKp30-dependent [38, 39] (Supplementary Figure 3B). Lack of staining of NKp30-Fc soluble receptor on MMECs might be due to the poor surface expression of B7-H6, not sufficient to bind NKp30-Fc soluble receptor, but capable of engaging the native NKp30 receptor. Alternatively, both MMECs and iDC may express an additional, not yet identified, NKp30 ligand that is not recognized by the NKp30-Fc soluble receptor.

### rIL-27 plus suboptimal doses of rIL-15 enhance NK cell cytotoxicity against tumor endothelial cells

**Figure 3:**
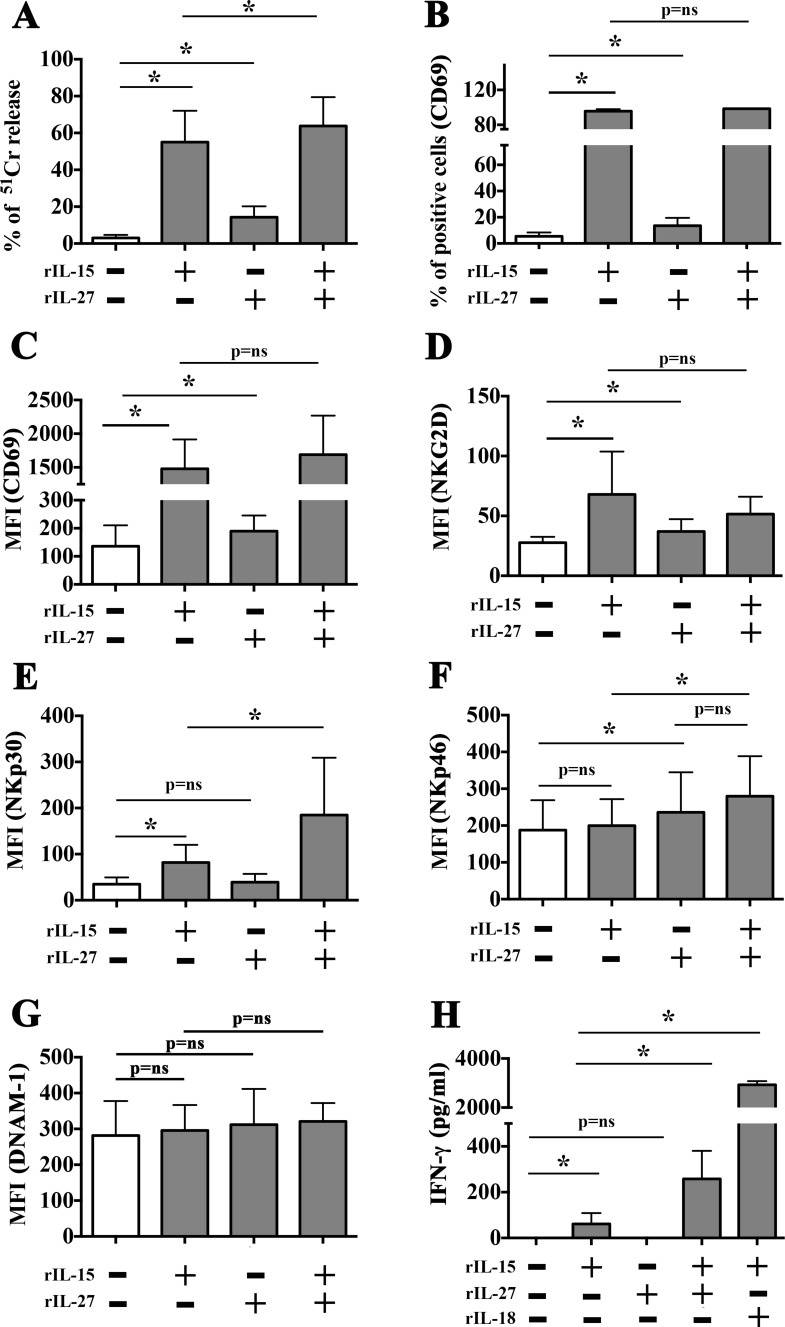
Comparison between rIL-27 and rIL-15-mediated stimulation of resting NK cells NK cells purified from healthy donors were stimulated with rIL-15 (20 ng/ml) and rIL-27 (100 ng/ml) used alone or in combination. Cells were analyzed for (**A**) cytotoxicity against EA cells (^51^Cr release assay, E:T ratio 20:1), (**B**–**G**) expression of the indicated surface molecules (flow cytometry). Culture supernatants were analyzed for the presence of IFN-γ (ELISA assay) (**H**). Mean and 95% confidence intervals are indicated. *p < 0.05, *p* = ns means not significant. Data shown are pooled from 6 independent experiments performed using NK cells from 6 unrelated healthy donors.

NK cells stimulated with optimal doses of rIL-15 displayed cytotoxicity against all MM-associated endothelial cells analyzed, an effect that was accompanied by the upregulated expression of CD69 activation marker (Figure 3B and 3C), NKG2D (Figure 3D) and NKp30 (Figure 3E) and by a modest but significant production of IFN-γ (Figure 3H). However, IL-15 might exert unwanted *in vivo* side effects due to its strong pro-angiogenic activity. Thus, we analyzed the function of NK cells cultured in the presence of rIL-27 (100 ng/ml), which has been described to combine immunostimulatory and anti-angiogenic properties. As shown in Figure 3, the stimulatory capability of rIL-27 was lower than that of rIL-15. In particular, rIL-27 induced a slight increase in NK cell-mediated killing of EA cells (Figure 3A), a modest increase in the surface expression of CD69 and NKG2D, and did not stimulate the release of IFN-γ. It is of note, however, that rIL-27 caused a peculiar upregulation of the NKp46 receptor (Figure 3F). When used in combination with rIL-15, rIL-27 did not improve CD69 and NKG2D upregulation but significantly increased the cytolytic activity, the surface expression of NKp30 and the IFN-γ production as compared to rIL-15 alone (Figure 3). DNAM-1 expression was not modified by rIL-27 or rIL-15 used either alone or in combination (Figure 3G).

**Figure 4:**
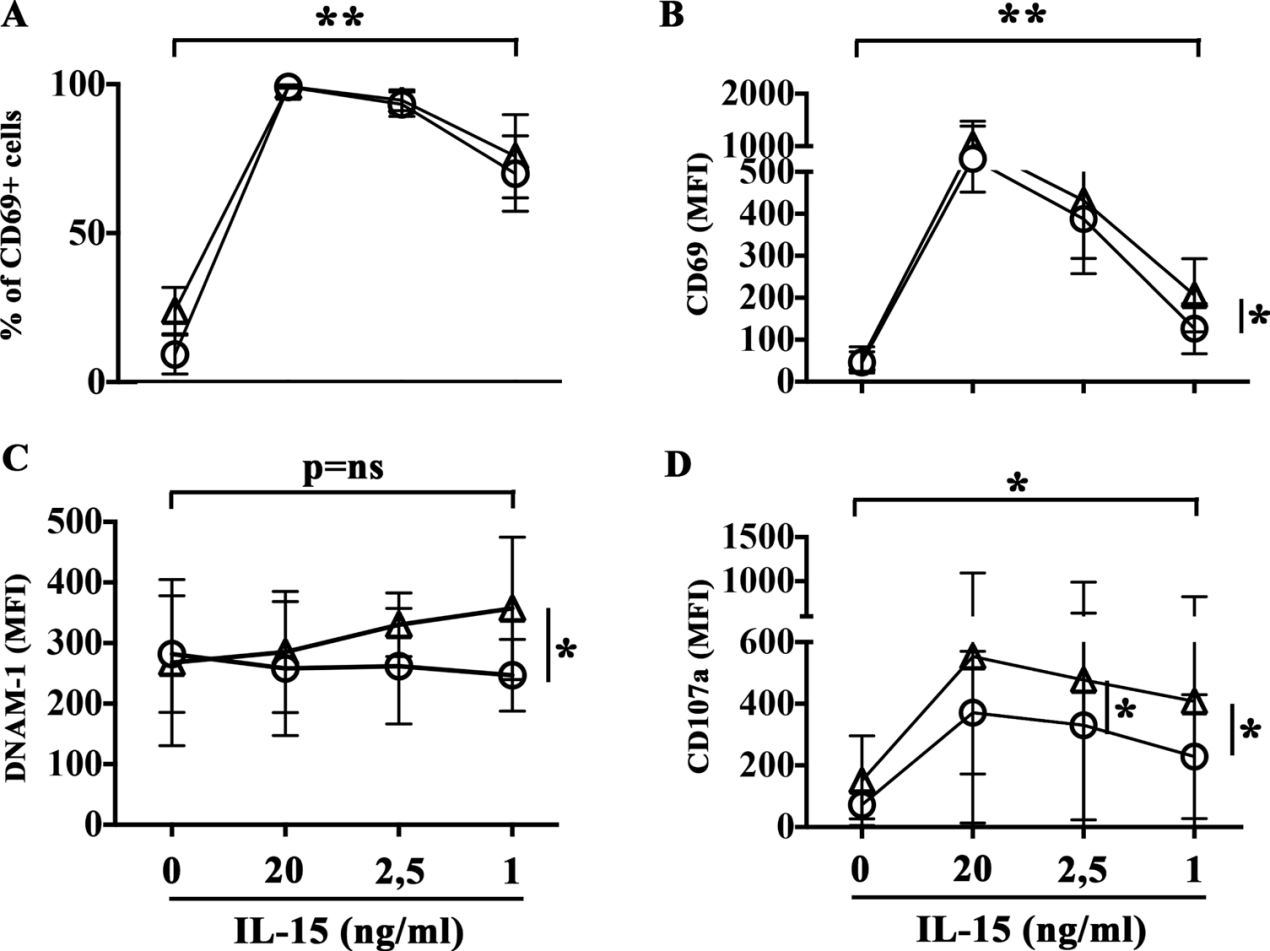
rIL-27 plus suboptimal doses of rIL-15 induce NK cell activation NK cells purified from healthy donors were stimulated with decreasing concentrations of rIL-15 alone (circle) or in combination with rIL-27 (100 ng/ml) (triangle). Cells were analyzed for (**A**–**C**) surface phenotype (flow cytometry), (**D**) release of cytotoxic granules in the presence of EA cells (CD107a assay). Mean and 95% confidence intervals are indicated. *p < 0.05, **p < 0.01. *p* = ns means not significant. Data shown are pooled from 6 independent experiments performed using NK cells from 6 unrelated healthy donors.

**Figure 5:**
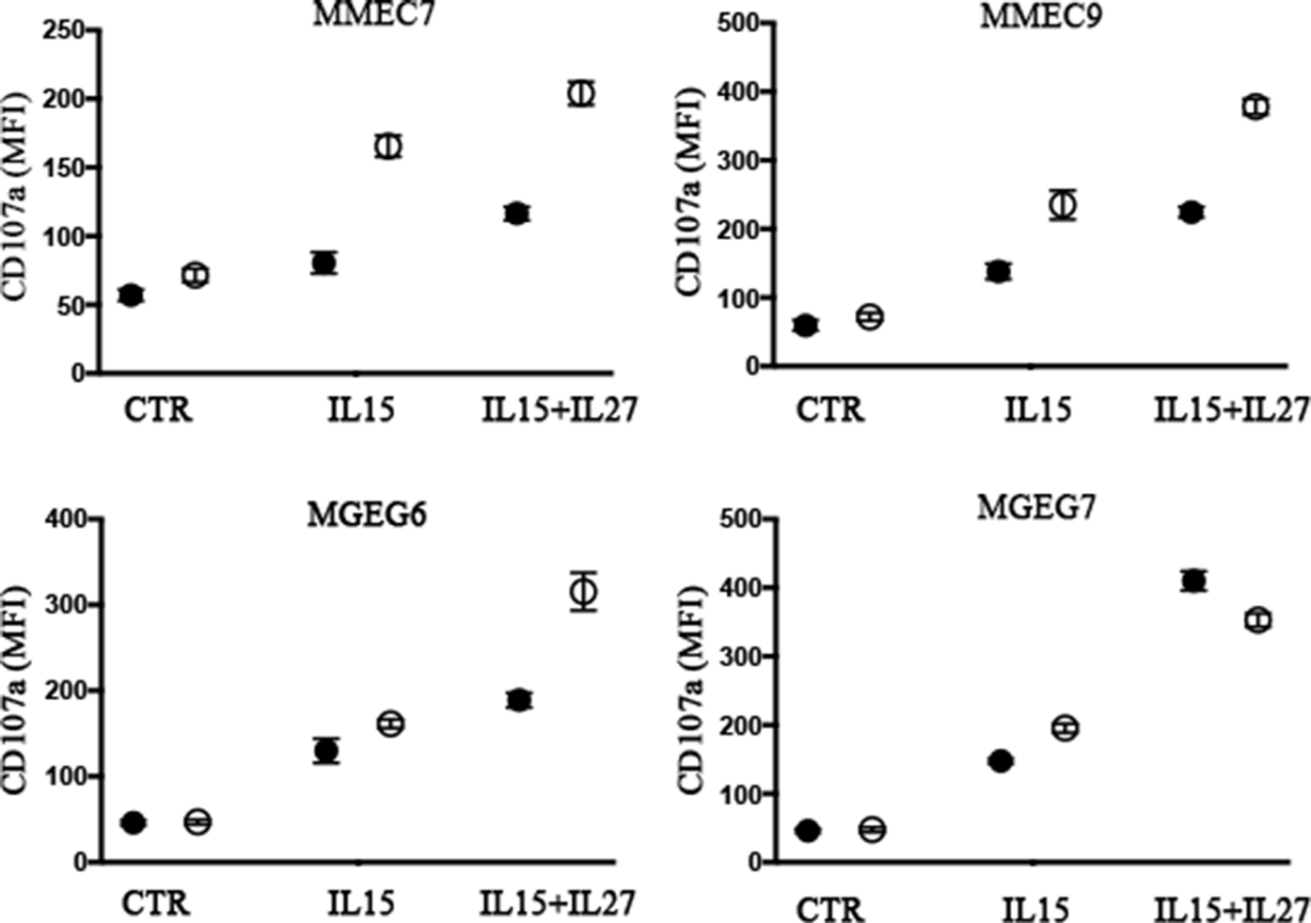
NK cell degranulation in the presence of MMECs and MGECs is increased by rIL-27 and suboptimal concentrations of rIL-15 NK cell populations derived from 2 healthy donors (black and white circles) were stimulated with suboptimal concentrations (1 ng/ml) of rIL-15 alone or in combination with rIL-27 (100 ng/ml). Cells were analyzed for degranulation (CD107a assay) in the presence of the indicated MMECs and MGECs. Controls (CTR) represent NK cell degranulation of NK cells stimulated with rIL-15 and rIL-27 in the absence of target. Data shown are pooled from 2 independent experiments. Mean are indicated.

To determine the dose of cytokines suitable to induce NK cell activation minimizing pro-angiogenic side effects, NK cells were treated with rIL-27 in combination with decreasing concentration of rIL-15, ranging from 20 to 1 ng/ml (Figure 4). NK cells stimulated with rIL-27 and the lowest rIL-15 concentration used (1 ng/ml) showed a significant increased expression of CD69 (in terms of both % of positive cells and MFI) and levels of degranulation in the presence of EA cells that reached those observed in the presence of optimal doses of rIL-15. Moreover, rIL-27 promoted an upregulation of DNAM-1 expression (Figure 4C) that progressively increased by reducing the concentration of rIL-15. The ability of rIL-27 to increase NK cell degranulation in the presence of suboptimal doses of rIL-15 (1 ng/ml) was confirmed using as targets both MMECs and MGECs (Figure 5).

### rIL-27 up-regulates PD-L2 and HLA class I expression in tumor endothelial cells

We analyzed whether the positive effects of the rIL-15^1 ng^-rIL-27^100 ng^ combination on NK cell functions could be associated with side effects such as the up-regulation of ligands capable to inhibit the NK cell-mediated attack.

**Figure 6:**
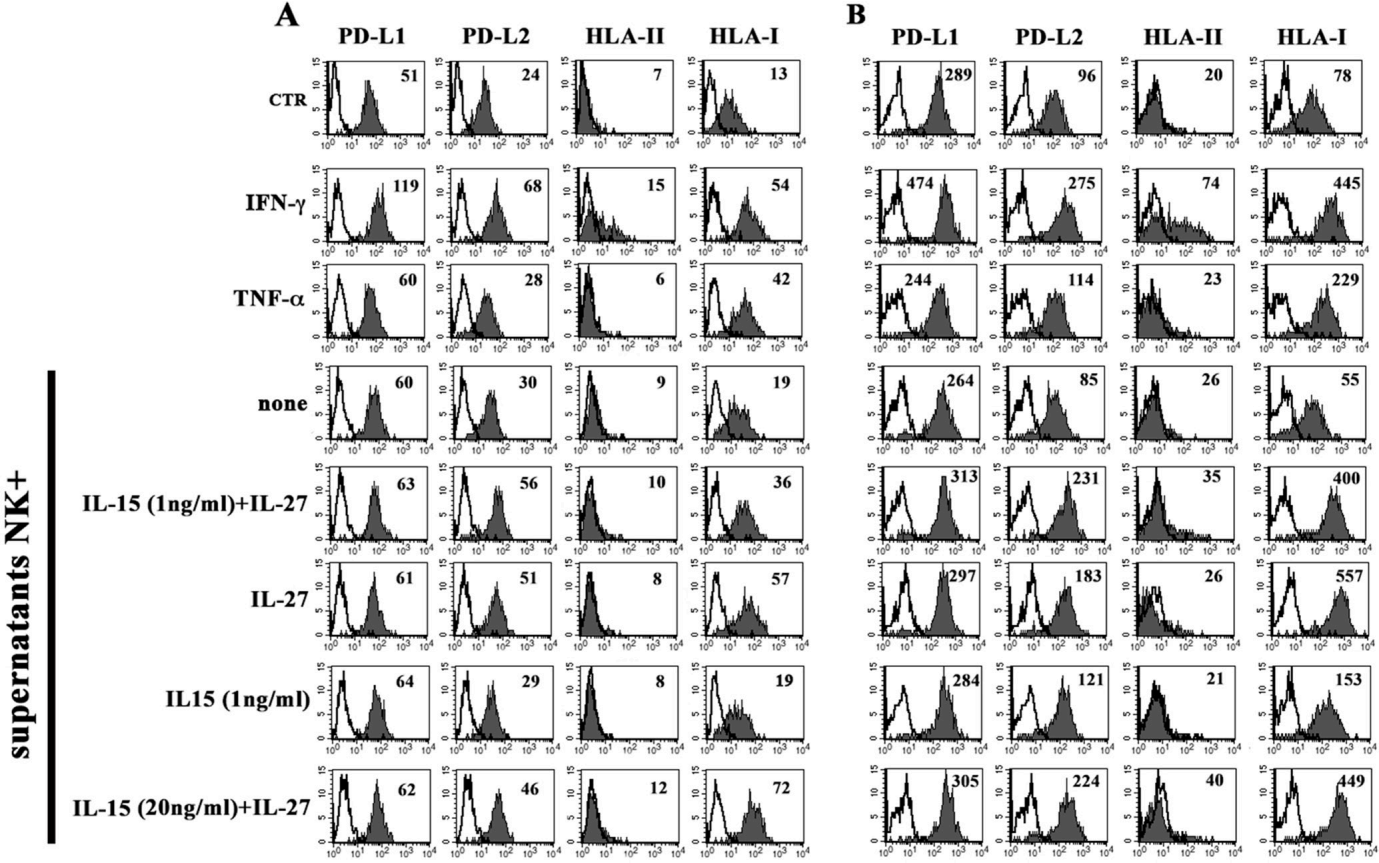
Constitutive or inducible expression of PD-Ls, HLA-II and HLA-I in EA cell line The EA cell line untreated (CTR), treated with IFN-γ, TNF-α or culture supernatants from NK cells unstimulated (none) or stimulated with the indicated cytokines, was analyzed by flow cytometry for the expression of PD-Ls, HLA-II and HLA-I. Panel **A** and **B** show two independent experiments performed using NK cells purified from two unrelated healthy donors. White profiles refer to cells incubated with isotype-matched mAbs. Values inside each histogram indicate the MFI.

The EA cell line was analyzed for the expression of the PD-Ls and HLA-II immune-checkpoints molecules, as well as of HLA-I. As shown in Figure 6, EA cells constitutively expressed PD-L1, PD-L2 and HLA-I. Similar to what has been demonstrated in tumor cells [34, 40], rIFN-γ up-regulated the PD-Ls and HLA-I expression and de novo induced that of HLA-II, whereas rTNFα was effective only in the induction of HLA-I expression.

**Figure 7:**
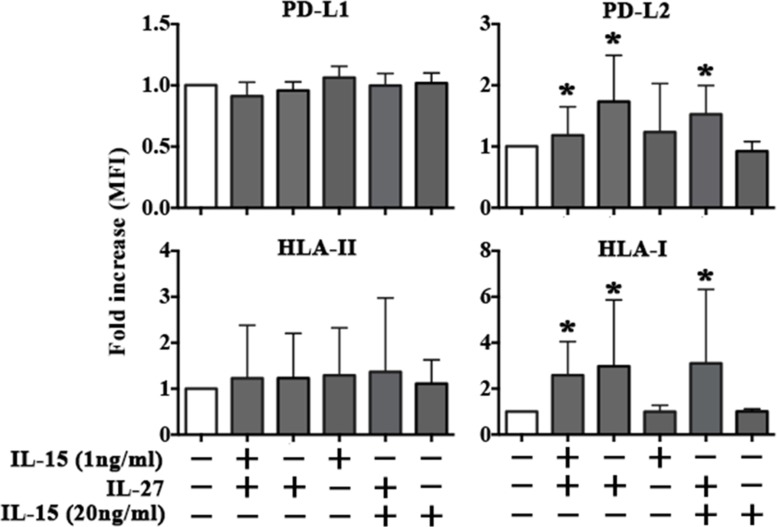
rIL-27 upregulates PD-L2 and HLA-I in EA cell line The EA cell line untreated (white bar) or treated with rIL-15 and rIL-27 alone or in combination (gray bars) was analyzed by flow cytometry for the expression of PD-Ls, HLA-II and HLA-I. MFI fold increase is shown. Mean and 95% confidence intervals are indicated. *p < 0.05. Data shown are pooled from 4 independent experiments.

EA cells were treated with culture supernatants derived from NK cells stimulated with the rIL-15^1 ng^-rIL-27^100 ng^ combination. EA cells did not modify the expression of PD-L1 and HLA-II, whereas they showed upregulation of PD-L2 and HLA-I (Figure 6) at levels comparable to those obtained using rIFN-γ. It is of note that a similar effect was obtained using supernatants from NK cells stimulated with rIL-27^100 ng^ but not with rIL-15^1 ng^ (Figure 6). Moreover, negligible amounts of IFN-γ were detected in the supernatants of NK cells stimulated with rIL-15^1 ng^ and rIL-27^100 ng^, both used alone or in combination (Supplementary Table 1). Overall the data suggested that the increased expression of PD-L2 and HLA-I in EA cells treated with NK supernatants was mainly rIL-27 dependent and IFN-γ/IL-15 independent. To confirm this hypothesis, EA cells were directly stimulated with the cytokines either alone or in combination (Figure 7 and Supplementary Figure 4). rIL-27 alone did not modify the expression of PD-L1 and HLA-II, whereas increased that of PD-L2 and HLA-I that reached levels comparable to those obtained using the rIL-15^1ng^-rIL-27^100 ng^ combination. On the contrary, rIL-15 alone had no effect even at the highest (20 ng/ml) concentration used.

**Figure 8:**
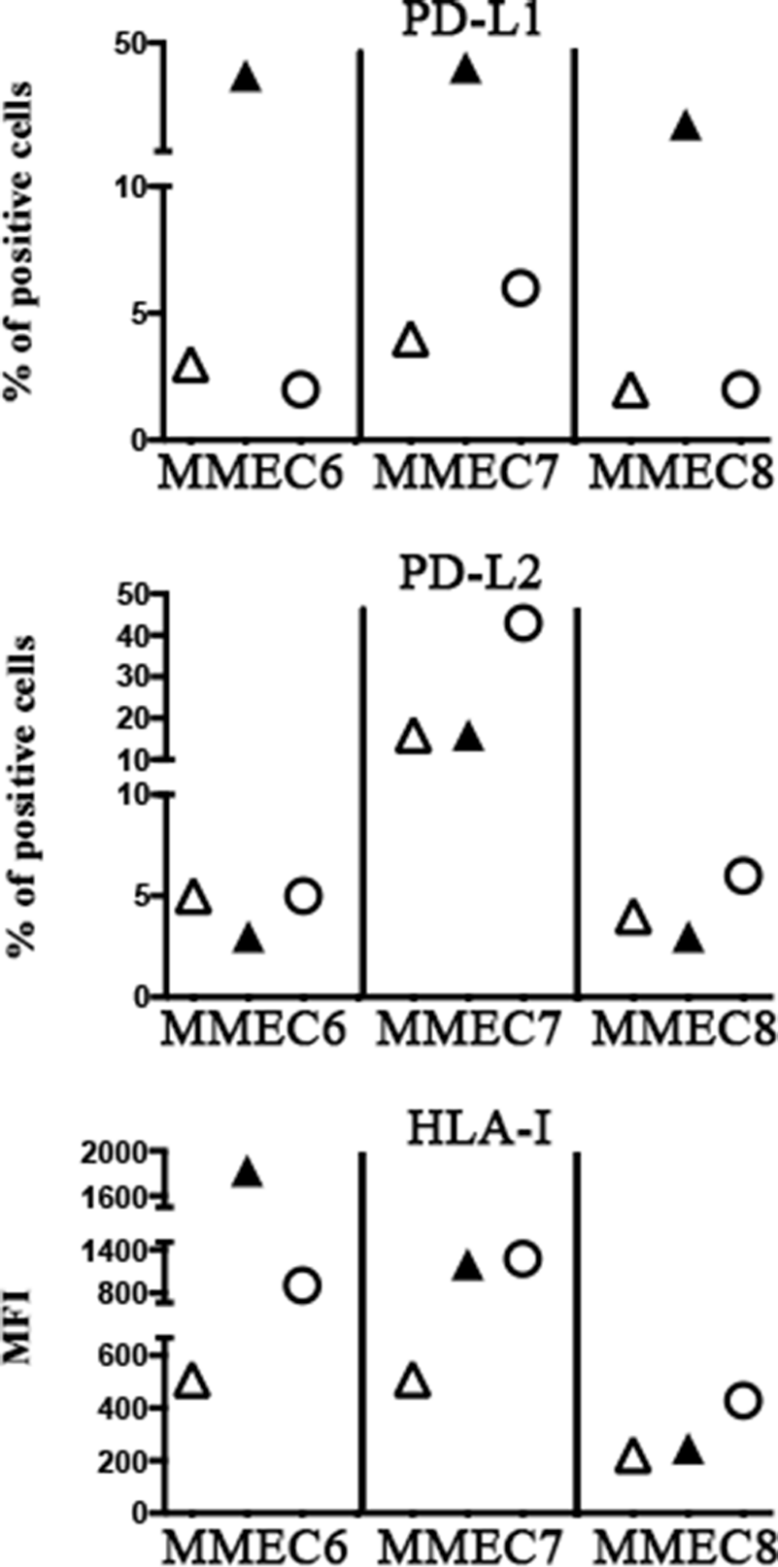
Effect of rIL-27 on PD-Ls and HLA-I expression in MMECs Three MMECs either untreated (open triangle) or treated for 5 days with IFN-γ (black triangle) or IL-27 (open circle) were analyzed by flow cytometry for the expression of PD-Ls and HLA-I. Percentage of positive cells or MFI is indicated.

The rIL-27 capability of up-regulating HLA-I and PD-L2 molecules in tumor endothelium was confirmed using three different MMECs that were treated with rIL-27^100 ng^ (or IFN-γ, as control) (Figure 8). According to results obtained on EA, rIL-27 did not significantly modify the PD-L1 expression whereas it up-regulated HLA-I expression in all MMECs, although at different extent. Increased PD-L2 expression was detected in MMEC7 that derived from a patient with MM in progression and unresponsive to chemotherapy. In line with results on EA, IFN-γ induced PD-L1 and HLA-I expression on MMEC6 and MMEC7. Interestingly however, on MMEC8 derived from a patient in relapse IFN-γ increased PD-L1 but not HLA-I expression.

## DISCUSSION

Multiple Myeloma remains an incurable disease and novel therapeutic strategies did not lead to a survival higher than 5 years in the adult [41]. Some benefits have been achieved using drugs such as lenalidomide that can exert several immunomodulatory effects, including the exacerbation of NK cell-mediated cytotoxicity against MM [42]. *In vitro* studies and clinical trials have also explored the possible efficacy of novel strategies combining standard treatments with emerging therapies aimed to interrupt receptors/ligands interactions such as PD1/PD-L1 [43] or KIRs/HLA-I [44], which are capable of limiting the anti-tumor function of immune effectors. NKp46, NKG2D and DNAM-1 activating receptors recognize specific ligands on MM cell surface and unchain the anti-MM activity of NK cells [45, 46]. In particular, as for other malignancies [21, 22], a pivotal role in the NK-mediated aggression of MM is played by DNAM-1/PVR interactions. These are required for optimal anti-MM efficacy of standard therapies based on the administration of cyclophosphamide and bortezomib. Moreover, it has been shown that different classes of therapeutic agents upregulate DNAM-1 as well as NKG2D ligands [47, 48], thus strengthening the concept that NK cells might truly represent powerful adjuvant arms against MM.

Our present study suggests that NK cell-based immunotherapy might also be effective in MM patients because of the capability of NK cells to kill tumor-associated endothelial cells, which are involved in neo-angiogenesis and represent a source of soluble factors involved in paracrine loops mediating plasma cell proliferation and spread [49, 50]. We showed that activated NK cells efficiently killed MMECs (and MGECs) thanks to the cooperation of multiple triggering receptors. These include NKp30 whose known ligand B7-H6, however, is virtually absent on tumor endothelium, suggesting the possible existence of novel unidentified NKp30 specific ligand/s. NKG2D ligands were weakly expressed on MMECs surface, whereas all MMECs analyzed expressed good levels of both PVR and Nectin-2, ligands of DNAM that clearly contributed to killing of MMECs. A striking exception was represented by MMEC1 and MMEC2 that resulted poorly susceptible to lysis despite the expression of high levels of PVR. DNAM-1/PVR interaction is crucial for recognition and killing of different tumors [3]. It is of note however that its action could be counteracted by the engagement of TIGIT [20, 51] an inhibitory receptor which recognizes nectin-3 as well as PVR and nectin-2, with higher affinity than DNAM-1 [51]. Thus the different susceptibility to killing of PVR^high^ MMECs might be linked to different combinations of PVR-specific paired receptors on NK cells [51]. Another possibility is that in some patients the function of TIGIT may dominate due to the expression of nectin-3 on MMECs. Both hypotheses are currently under investigation.

In line with data obtained from the immunohistochemical analysis of primary glioblastoma specimens [8], PVR showed a high expression in tumor-associated endothelium from MM BM aspirates. In a physiological context, DNAM-1/PVR interactions have been shown to promote the transendothelial migration process of monocytes [52]. It would be of interest to understand whether the high expression of PVR in MMECs might improve migration of monocytes, which in the tumor microenvironment tend to differentiate into macrophages and acquire a M2 tumor-promoting functional polarization [53, 54]. Moreover, PVR expression might directly favor the function of MMECs thus contributing to the formation of the complex architecture of tumor vasculature. In this context, different studies showed that PVR improve tumor cell invasion, being localized at the migrating cellular front together with actin and alphav-integrin, known mediators of motility and adhesion [55–57]. Thus, it should be taken into consideration that therapies upregulating PVR expression could on one side potentiate the NK cell-mediated killing by improving the formation of effective immune synapses, on the other they may favor functional capabilities of MM and MMECs.

DNAM-1/PVR interactions also occur during the crosstalk between NK cells and Dendritic cells (DC) [58, 59] or Macrophages [60, 61]. During their activation these antigen presenting cells (APC) release different cytokines capable of amplifying NK (and T) cell-mediated responses. These include IL-27 that is of interest in tumor immunology because it combines immunostimulatory and anti-angiogenic properties [30, 31]. We have analyzed the effects of IL-27 on NK cell functions, using the cytokine alone or in combination with IL-15. IL-15 represents a promising immunostimulatory adjuvant for therapies [62, 63], and a clinical grade formulation is currently available. However, different data suggested that IL-15 is also endowed with strong pro-angiogenic effects [64, 65]. According to previous data, we showed that optimal doses of IL-15 efficiently stimulate NK cells and increase their killing of MMECs. Interestingly, a similar cytolytic potential was observed in NK cells stimulated with IL-27 and suboptimal doses of IL-15 (IL-15^1 ng^ and IL-27^100 ng^). This effect was paralleled by a trend toward the increased expression of DNAM-1, NKp30, NKG2D and NKp46 activating receptors. In this context, and according with previous published data [66], the upregulation of NKp46 in NK cells was mainly IL-27-dependent.

In some instances, IL-27 did not ameliorate the effect of optimal doses of IL-15. For example, a significant increase of DNAM-1 expression could be appreciated only when IL-27 was used in combination with suboptimal doses of IL-15 (see Figures 3 and 4). This observation could be partially due to the fact that, in NK cells, IL-15- and IL-27-mediated signals share some transducing molecules such as JAK1 and STAT5 [67, 68]. Moreover, IL-15 might play a role in regulating IL-27R expression and/or function (under investigation).

NK cells stimulated with IL-27 and suboptimal doses of IL-15, release negligible amounts of IFN-γ, which is considered the best inducer of HLA-I molecules as well as of the PD-Ls immune-checkpoints ligands [34]. In EA and MMECs, IL-27 (either alone or in combination with IL-15^1ng^) did not upregulate the expression of PD-L1 or induce that of HLA-II. On the other hand, it upregulated the expression of HLA-I, as previously demonstrated in normal endothelium (HUVEC cells) [69]. Moreover our study highlighted a peculiar function of this cytokine, i.e. the capability of upregulating the expression of PD-L2 on tumor endothelium. It is of note that previous published data suggest that IL-27 function might vary depending on target cell type and cytokine milieu. Indeed, IL-27 has been shown to drive upregulation of HLA-I also in chronic eczema keratinocytes that produced IL-27, but it promoted PD-L1 expression in different cell types including CD4+ and CD8+ T cells, monocytes, DC and tumor cells [29, 30, 70]. Thus, similar to IFN-γ (and TNF-α), IL-27 might exert possible side effects modulating the expression in MMECs of HLA-I, which downregulates the NK cell function, and PD-L2 that controls the duration and amplitude of both CD8+ T and NK cell functions. In this context, it has been shown that in MM patients more than 50% of peripheral blood NK cells express PD-1 [43], the PD-Ls receptor, although at levels lower than PD1^+^ NK cells detected in normal individuals or in different pathological conditions [72]. Thus, standard or emerging therapeutic approaches should not disregard the possible induction of a cytokine storm that might shape immune responses against tumor cells and/or tumor-associated endothelium. As for IFN-γ [71] the IL-27 capability of upregulating immune checkpoint ligands on tumor endothelium, may greatly vary among patients. Notably, during anti-tumor immune responses IL-27 may act earlier than IFN-γ, being a cytokine produced by innate cells such as macrophages and DC.

## MATERIALS AND METHODS

### Patients

Bone marrow (BM) aspirates were obtained from nine MM patients fulfilling the International Myeloma Working Group diagnostic criteria for multiple myeloma (MM). MM patients, enrolled at diagnosis (MM1-6, MM9), in progression because unresponsive to therapy (MM7), in relapse (MM8), were characterized by active, symptomatic disease (D&S stage II-III). Additional BM aspirates were obtained from a MM patient in complete/objective remission (cr-MM), seven patients with monoclonal gammopathy of undetermined significance (MGUS), and a patient with anemia due to iron deficiency (IDA). Approval from the Ethics Board was obtained (N°4220/2013), and patients were asked to provide their written informed consent in accordance with the Declaration of Helsinki.

### Cells used in the study

Primary endothelial cells (ECs) were obtained from BM of patients as described [37]. Briefly, ECs were purified from BM-derived mononuclear cells by immunoselection, using magnetic microbeads (Dynal, Oslo, Norway) coated with Ulex europaeu*s* agglutinin-1 lectin (UEA-1, Sigma Chemical), whose receptor is selectively and highly expressed by endothelial cells. ECs bound to microbeads were cultured in complete DMEM medium supplemented with 20% of heat-inactivated fetal bovine serum (FBS) to allow cells to adhere, spread and grow. Endothelial cells were growth for at least one passage and their purity was confirmed by flow cytometry (FACScalibur, Becton Dickinson and Co, Mountain View, CA) analyzing the presence of the ECs markers factor VIII–related antigen (anti-human Von Willebrand factor mAb, Beckman Coulter, Marseille, France) and CD105 (anti-human CD105 mAb, Beckman Coulter), and the absence of CD14 (anti-human CD14 mAb, Becton Dickinson) and CD38 (anti-human CD38 mAb, Becton Dickinson) molecules. ECs viability was assessed by trypan blue exclusion staining (> 97% viable cells). The human endothelial (EA.hy926, henceforth named EA) and erythroleukemia (K562) cell lines were purchased from American Type Culture Collection (ATCC).

After approval by the Ethics Board (N°39/2012) and informed consent, buffy coats were obtained from healthy volunteer blood donors admitted at the transfusion center of IRCCS AOU San Martino-IST (Genova, Italy). Peripheral blood mononuclear cells (PBMC) were isolated on Ficoll-Hypaque gradients and frozen at –80°C. Upon arrival of MMECs in the laboratory by express courier, PBMC were thawed and either stimulated with cytokines or used to purify NK cells (Human NK Cell Isolation kit, Miltenyi Biotec, GmbH, Bergisch Gladbach, Germany) as previously described [53]. The degree of purity of the isolated NK cells (CD3^-^, CD56^+^, NKp46^+^) was superior to 98%.

### Monoclonal antibodies and cytokines

The following mAbs were produced in our laboratory: A6136 (IgM) and 6A4 (IgG1) (anti-HLA class I-A, -B, -C and HLA-E), FST24 (IgG2b, anti-HLA-II), A6/220 (IgM, anti-CD56), BAB281 (IgG1) and KL247 (IgM) (anti-NKp46), AZ20 (IgG1) and F252 (IgM) (anti-NKp30), BAT221 (IgG1, anti-NKG2D), KRA236 (IgG1) and F5 (IgM) (anti-DNAM-1), BAM195 (IgG1, anti-MICA), M5A10 (IgG1, anti-PVR), U191 (IgM, anti-Nectin-2), c227 (IgG1, anti-CD69). MAB1380 (IgG2a, anti-ULBP1), MAB163903 (IgG2A, anti-ULBP2), MAB1517 (IgG2A, anti-ULBP3) and M475 (IgG2B, anti-ULBP4) mAbs were purchased from R&D System Inc., (Minneapolis, MN, USA); anti-CD107a-PE and anti-CD56-PC5 mAbs were purchased from Becton Dickinson. Anti-PD-L1.3.1 (IgG1, anti-PD-L1) and anti-PD-L2 (IgG1, anti-PD-L2) were generated in D. Olive's lab.

Human recombinant cytokines were purchased from PeproTech (rIL-15, rIFN-γ and rTNFα) MBL International (rIL-18) and R&D Systems (rIL-27).

### Flow cytometry, cytolytic and ELISA assays

For one-color immunofluorescence and cytofluorimetric analysis (FACSCalibur Becton Dickinson) cells were stained with the appropriate mAbs or isotype matched controls followed by PE-isotype-specific goat anti-mouse second reagent (Southern Biotechnology Associated, Birmingham, AL). On every experimental session, the flow cytometer performances were monitored and the reproducibility of the fluorescence intensity was aligned by calibrated microsphere (Becton Dickinson). ECs were gated on the basis of physical parameters (SSC = Side Scatter; FSC = Forward scatter) to detect viable cells. To verify the appropriateness of the gating strategy cells were stained (10 minutes at the room temperature) with Annexin V and To-Pro-3 Iodide (Life Technologies, CA, USA).

Purified NK cells were cultured for 2 days in the presence of recombinant cytokines to obtain polyclonal activated NK cell populations, which were analyzed for their cytolytic activity against target cells using a 4 h ^51^Cr-release assay [21]. For CD107a (LAMP-1) degranulation assay NK cells were incubated for three hours with target cells in the presence of a PE-conjugated anti-human CD107a (IgG1; BD Biosciences).

The IFN-γ enzyme-linked immunosorbent human assay (ELISA) was performed according to the manufacturer's instruction (Life Technologies).

### Cell transfectants and chimeric receptors

The BW5147/B7-H6+ stable cell transfectant was prepared by retrovirus gene transfer using a B7-H6 ORF cDNA obtained by RT-PCR from the MM6 human myelomonocytic cell line and subcloned in pMXs-IG (IRES-GFP) retrovirus vector (kindly provided by Dr. Kitamura, Tokyo, Japan). The empty vector pMXs-IG was used to generate the negative control (mock cell transfectant). DNAM1-Fc and NKp30-Fc were obtained in our lab fusing the extracellular region of the receptor with a mutated human IgG1 Fc portion that lack the ability to bind FcRs [52, 73].

### Real-time PCR

Total RNA was extracted from K562 and EA cell lines and from immature dendritic cells (iDC) using RNAeasy Mini Kit (Qiagen, Hilden, Germany) according to manufacturer's instructions. Starting from 1 μg RNA, oligodT-primed cDNA was prepared using Transcriptor First Strand Synthesis Kit (Roche diagnostic, Mannheim, Germany). The expression of B7-H6 and HPRT1 (hypoxanthine phosphoribosyltransferase 1) transcripts was assessed by real-time PCR with the Taqman Gene expression assays (Hs02340611_m1 and Hs99999909_m1, respectively) and Express qPCR SuperMix (Thermo Fisher Scientific, Waltham, MA, USA). Samples were run on a ViiA 7 Real-Time PCR System (Thermo Fisher Scientific). Each reaction was performed in triplicate and each sample was analysed in three independent experiments. Relative expression of B7-H6 transcript was determined in each sample by normalization with respect to the HPRT1 gene, according to the standard ΔC_T_ method.

### Statistical analysis

Statistical analysis with level of significance (p) and graphic representation were performed using Wilcoxon-Mann-Whiteny *p-value* test (non-parametric significance test) and GraphPad Prism 6 (GraphPad Software La Jolla, CA).

## SUPPLEMENTARY MATERIALS FIGURES AND TABLES


