# Supplementary material for: NK cells and multiple myeloma-associated endothelial cells: molecular interactions and influence of IL-27
Source: Oncotarget. 2017 Apr 12;8(21):35088–102. doi: 10.18632/oncotarget.17070 (PMC5471037; doi:10.18632/oncotarget.17070)
Supplement: Supplementary file 1 [file oncotarget-08-35088-s001.pdf]

NK cells and multiple myeloma-associated endothelial cells: molecular interactions and influence of IL-27

Supplementary Materials

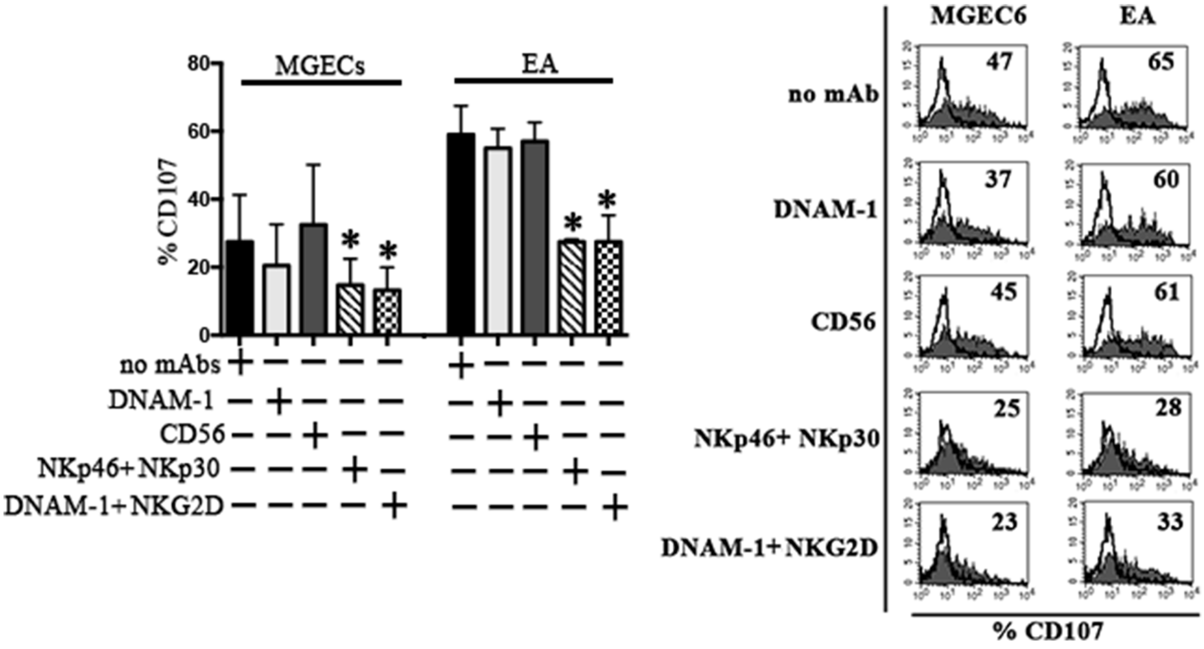

Supplemental Figure 1: Activating receptors involved in NK cell degranulation in the presence of MGECs. (**A**) IL-15 activated NK cells were analyzed for degranulation (CD107a assay) in the presence of MGECs and EA cell line (E:T ratio 1:1), either in the absence (no mAb) or in the presence of mAbs (10 µg/ml) specific for the indicated activating NK receptors, used alone or in combination. Mean, 95% confidence intervals and significance are indicated. $*p < 0.05$. Data shown are pooled from 3 independent experiments performed using NK cells purified from 3 unrelated healthy donors. (**B**) Representative cytofluorimetric analysis of NK cell degranulation in the presence of MGECs (MGEC6) and EA cell line, with or without mAbs. In each histogram numbers indicate the % of $CD107^{+}$ cells.

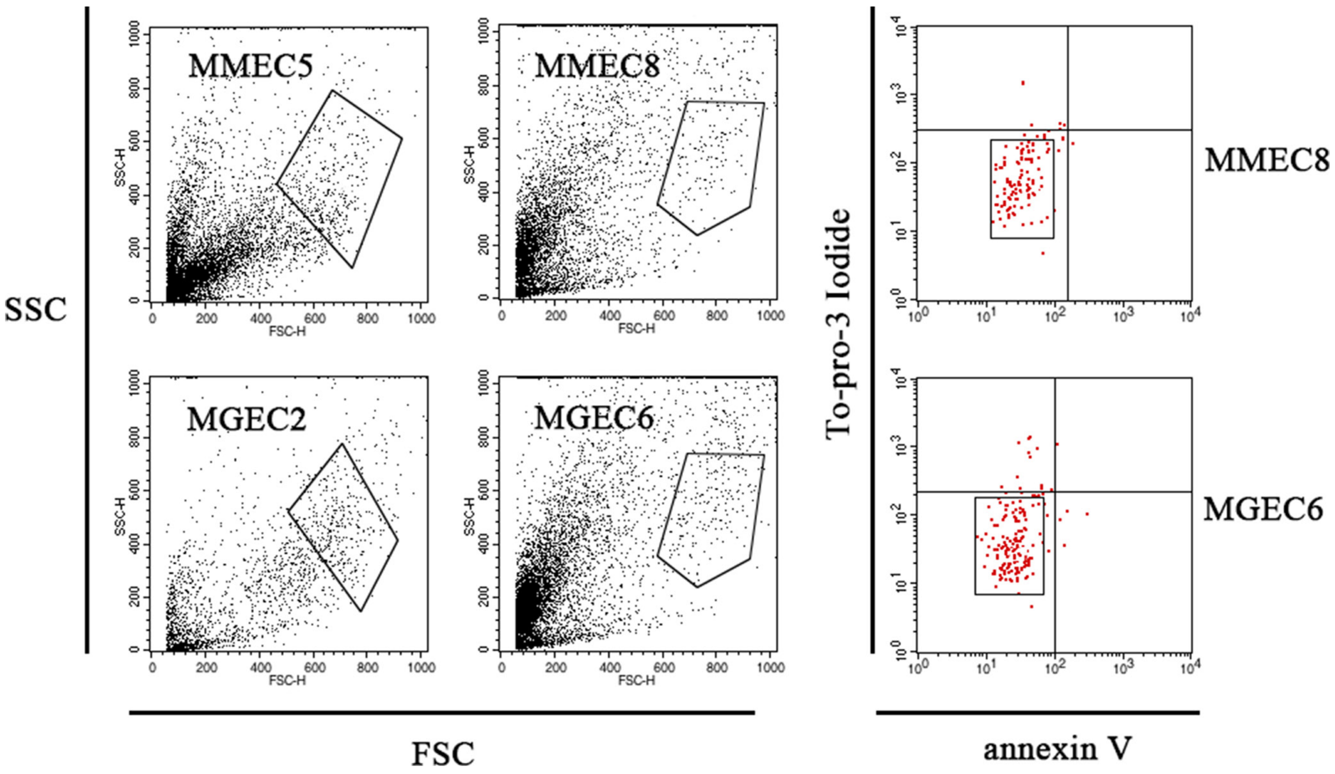

Supplemental Figure 2: MMECs and MGECs flow cytometry: gating strategy. MMECs and MGECs were gated on the basis of physical parameters (SSC = Side Scatter; FSC = Forward scatter). The percentage of gated viable cells (Annexin V and To-Pro-3 double negative cells) was > 95%.

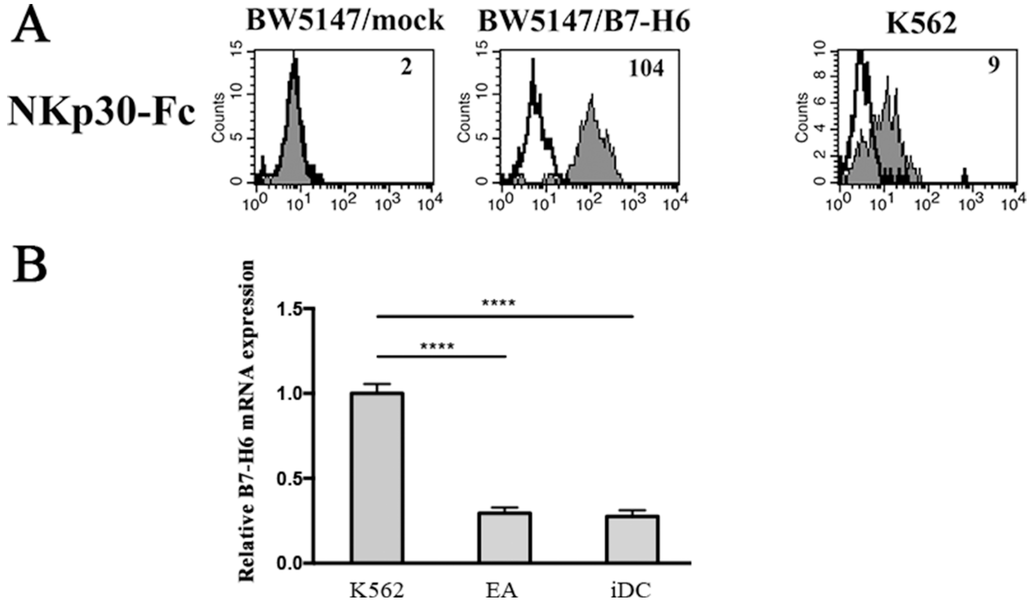

Supplemental Figure 3: Reactivity of NKp30-Fc molecule in B7-H6+ cells and B7-H6 mRNA expression on in EA endothelial cell line. (**A**) Representative cytofluorimetric analysis of NKp30-Fc reactivity in mock or B7-H6 transfected BW5147 cell line and K562 cell line. White profiles refer to cells incubated with a control Receptor-Fc molecule. Values inside each histogram indicate the MFI. (**B**) B7-H6 mRNA expression in K562, EA cell line and immature DCs. The levels of B7-H6 mRNA in K562 cell line were chosen as reference and were arbitrarily normalized to 1. Data shown are pooled from 3 independent experiments analysed in triplicate. Standard error is indicated (*****p* < 0,0001).

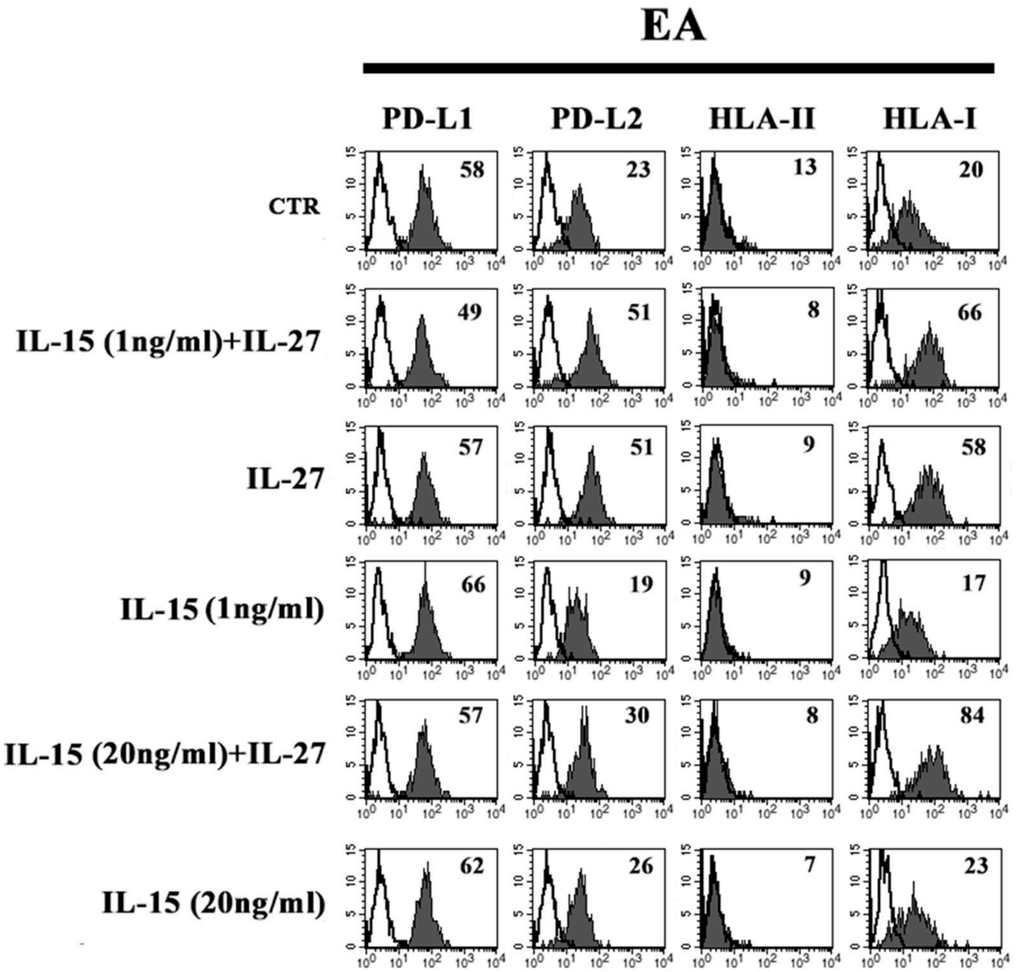

Supplemental Figure 4: rIL-27-mediated upregulation of PD-L2 and HLA class I in tumor endothelium. Representative cytofluorimetric analysis of immune-checkpoints and HLA-I expression in EA cells untreated (CTR) or treated with the indicated cytokines combinations. White profiles refer to cells incubated with an isotype-matched mAb. Values inside each histogram indicate the MFI.

Supplementary Table 1: IFN-γ content in the supernatants of cytokine-stimulated NK cells

NK cell stimulation	**IFN-γ content (pg/ml)**	
	**NK from DONOR 1**	**NK from DONOR 2**
none	5	9
IL-15 (1 ng/ml) + IL-27 (100 ng/ml)	6	19
IL-27(100 ng/ml)	2	6
IL-15 (1 ng/ml)	13	48
IL-15 (20 ng/ml) + IL-27 (100 ng/ml)	130	401

NK cells from 2 healthy donors were stimulated with the indicated cytokines used alone or in combination. On day 2, culture supernatants were recovered and analyzed for the presence of IFN-γ (ELISA assay).
